# Loss of Fibrinogen in Zebrafish Results in Symptoms Consistent with Human Hypofibrinogenemia

**DOI:** 10.1371/journal.pone.0074682

**Published:** 2013-09-30

**Authors:** Andy H. Vo, Alok Swaroop, Yang Liu, Zachary G. Norris, Jordan A. Shavit

**Affiliations:** Department of Pediatrics and Communicable Diseases, University of Michigan, Ann Arbor, Michigan, United States of America; Emory University School of Medicine, United States of America

## Abstract

Cessation of bleeding after trauma is a necessary evolutionary vertebrate adaption for survival. One of the major pathways regulating response to hemorrhage is the coagulation cascade, which ends with the cleavage of fibrinogen to form a stable clot. Patients with low or absent fibrinogen are at risk for bleeding. While much detailed information is known about fibrinogen regulation and function through studies of humans and mammalian models, bleeding risk in patients cannot always be accurately predicted purely based on fibrinogen levels, suggesting an influence of modifying factors and a need for additional genetic models. The zebrafish has orthologs to the three components of fibrinogen (*fga*, *fgb*, and *fgg*), but it hasn’t yet been shown that zebrafish fibrinogen functions to prevent bleeding *in vivo*. Here we show that zebrafish fibrinogen is incorporated into an induced thrombus, and deficiency results in hemorrhage. An Fgb-eGFP fusion protein is incorporated into a developing thrombus induced by laser injury, but causes bleeding in adult transgenic fish. Antisense morpholino knockdown results in intracranial and intramuscular hemorrhage at 3 days post fertilization. The observed phenotypes are consistent with symptoms exhibited by patients with hypo- and afibrinogenemia. These data demonstrate that zebrafish possess highly conserved orthologs of the fibrinogen chains, which function similarly to mammals through the formation of a fibrin clot.

## Introduction

The coagulation cascade is a complex series of events culminating in the cleavage of fibrinogen by thrombin, resulting in formation of a fibrin clot and cessation of bleeding. A great deal of insight into the biology of these factors and the coagulation cascade itself has been achieved through the intersection of biochemical techniques and the study of individual patients with particular coagulation factor deficiencies [1], [2]. Human fibrinogen consists of 3 polypeptide chains, α, β, and γ, which are encoded by individual genes (*FGA*, *FGB*, and *FGG*, respectively) localized to a cluster on human chromosome 4. Inherited deficiencies of fibrinogen are rare, but result in either decreased levels (hypofibrinogenemia), absent fibrinogen (afibrinogenemia), or functional defects (dysfibrinogenemia). While fibrinogen is clearly one of the key proteins in hemostasis, symptoms can be relatively mild, even with severe deficiency [3], [4]. Despite the fact that fibrinogen functions downstream of factors VIII and IX, bleeding in afibrinogenemia generally appears to be less severe than hemophilia.

The laboratory mouse has been used as a robust tool to investigate coagulation factors and their modifiers, leveraging the large pedigree sizes and well established genetics of the model [5]–[10]. This includes a number of interesting studies which have utilized gene targeting of various fibrinogen chains (reviewed in [11]). However, one disadvantage of the mouse model is the cost of producing such large pedigrees. As an alternative approach, we and others have turned to the zebrafish model for hematologic studies [12]–[15] since the coagulation cascade is conserved in teleost fish [16]–[18]. The significant advantages of the zebrafish model include generation of 500–1500 offspring in the time it takes to produce 5–10 mice, and amenity to chemical mutagenesis and small molecule screening [19], [20].

While it has been demonstrated that zebrafish develop thrombosis in response to a laser-induced endothelial injury [21], and exhibit bleeding in response to knockdown of von Willebrand factor and prothrombin [22], [23], the functional role of the fibrinogen genes has not been directly evaluated. The presence of 3 syntenic orthologs (*fga*, *fgb*, and *fgg*) similar to human fibrinogen has been apparent from genomic sequence [24], and was recently proven by cytogenetic *in situ* hybridization [25]. In this study we show conservation of expression of zebrafish fibrinogens using *in situ* hybridization at 5 days post fertilization (dpf), complementing previous studies [25]. A fusion of green fluorescent protein (EGFP) with Fgb was incorporated into a developing thrombus induced by endothelial injury. Furthermore, loss-of-function analysis using morpholino antisense technology resulted in a bleeding diathesis with similarities to human patients with hypofibrinogenemia, demonstrating conservation of fibrinogen function.

## Materials and Methods

### Ethics Statement and Animal Use

Zebrafish (*Danio rerio*) were raised as described [26] in accordance with animal care guidelines as approved by the University of Michigan Animal Care and Use Committee under protocol number 10369. All animal care and use complied with the with the recommendations in the Guide for the Care and Use of Laboratory Animals of the National Institutes of Health. The transgenic line *Tg(gata1:dsred)* line was used for imaging of erythrocytes [27].

### In situ Hybridization

RNA *in situ* hybridization was carried out essentially as described with a few modifications [28]. Total mRNA was prepared from a single adult zebrafish using TRIzol (Invitrogen, Carlsbad, California). Total cDNA was synthesized with Superscript III reverse transcriptase after priming with random hexamers (Invitrogen). Sense and antisense probes were generated from cDNA using primers with SP6 and T7 overhangs (forward and reverse primers without overhangs, respectively for *fga* 5′-GCAAGTTTCCCACATCAGGT-3′ and 5′-GTCGGGCATATCTTCTTCCA-3′, *fgb* 5′-CAAGGAGTGCGAAGACATCA-3′ and 5′-GTATTTCCAGCCGTTCCTGA-3′, *fgg* 5′-TTGGACGTGGATGGACTGTA-3′ and 5′-GTGTCCCTGGAACTTGTCGT-3′), and transcribed *in vitro* with digoxygenin labeled nucleotides. Stained embryos or larvae were photographed using a Leica MXFLIII stereo fluorescent microscope with an Olympus (Tokyo, Japan) DP-70 digital camera. Embedding was in JB-4 resin as described [29], followed by sectioning at 4–6 µm using a Leica RM2265 ultra-microtome, and counter staining with eosin or nuclear fast red. Imaging of sections was with an Olympus BX-51 upright light microscope and Olympus DP-70 high resolution digital camera.

### Development of Liver-specific fgb-egfp Expressing Transgenic Zebrafish

pT2AL200R150G [30] (kindly provided by K. Kawakami) was digested with BamHI and BglII, and a 1 kb (kilobase) fragment containing *egfp* followed by an SV40 polyA signal sequence was isolated (there is a NcoI site just 3′ to the BamHI site, see below). pfabp10-CFP-NTRtol2 [31] was digested with BglII, releasing *cfp-ntr*, and ligated to the fragment from pT2AL200R150G, producing pfabp10-egfp. Oligonucleotides with 5′ NcoI recognition sites (5′-CCATGGctgaagtgtgagctctgcta-3′ and 5′CCATGGatttctgtttgaagtacggcc-3′) were used to amplify the full length zebrafish *fgb* cDNA by PCR, from the total cDNA prepared above. The 3′ primer was engineered such that the final amino acid of Fgb was included and fused to the initiation methionine of GFP. The 1.5 kb product was digested with NcoI and ligated into the NcoI site (site derived from pT2AL200R150G, noted above) of pfabp10-egfp, producing pfabp10-fgb-egfp. Polyadenylated transposase mRNA [32] was prepared using mMessage mMachine (Ambion, Austin, Texas) and 25 picogram (pg) of final circular plasmid was co-injected with 25 pg of transposase mRNA into either the cytoplasm or yolk of ABxTL hybrid one-cell embryos. F0 larvae were examined for hepatic fluorescence at 5 dpf using a Leica MZ16FA stereomicroscope. Several thousand F0 embryos were injected, identifying several hundred with hepatic fluorescence. ∼20 of the positive F0 individuals raised to adulthood were outcrossed to ABxTL. F1 offspring were screened for hepatic fluorescence and one line was identified with germline transmission.

### Induced Thrombosis in Zebrafish Larvae

Thrombi were induced by laser injury of the posterior cardinal vein (PCV) endothelium, essentially as described with some modifications [33]. At 5–6 dpf, *fabp10-fgb-egfp* transgenic larvae were anesthetized in 380 µM tricaine solution (Western Chemical Inc., Ferndale, WA) and embedded in 0.8% agarose on glass cover slips (24×55, Fisher Scientific, Waltham, MA). Larvae were placed on an inverted microscope (visualized with a 40× objective, Olympus IX71), and the PCV endothelium was targeted and ablated with a laser at the 5^th^ somite distal to the anal pore at maximum setting using 2 pulses, each lasting 5 seconds (MicroPoint Pulsed Laser System, Andor Technology, Belfast, United Kingdom). Images were acquired with a CCD camera (Olympus DP72) using cellSens software 1.3 (Olympus).

### Fibrinogen Knockdown and Analysis

Morpholino oligonucleotides (MOs) were ordered from GeneTools (Philomath, OR) to target the splice donor sites of *fga* (exon 1 5′-GCATTATATCACTCACCAATGCAGA-3′), *fgb* (exon 4 5′-ACTTTCTGAAGTGTAGTTACCCTCG-3′), and *fgg* (exon 3 5′-TTAATGATAAATTGTGCTACCTGGT-3′). MOs were injected into the yolk sac of 1–4 cell or cytoplasm of 1 cell embryos at injection solution concentrations of 0.5–1 mM. o-dianisidine staining was carried out as described at 2–3 dpf, after manual dechorionation if needed [34], [35]. For analysis of splicing, RT-PCR (reverse transcriptase-mediated PCR) was performed on pools of 10 injected embryos or larvae at 1 and 3 dpf, and uninjected embryos at 1 dpf using the following primers, forward and reverse, respectively, *fga* 5′-GGCAGAGAACGATGGTCAAT-3′ and 5′-TGGTCAGCTTTGTCCATGAG-3′, *fgb* 5′-AGCTGTGCAGGAGAAAGAGG-3′ and 5′-TAATGTTCTGGGGGAAGGTG-3′, *fgg* 5′-CACCATCACATGATTTGTGC-3′ and 5′-TGTTCTTCCTGAGCGAGAATG-3′, *elfa* 5′-CTTCTCAGGCTGACTGTGC-3′ and 5′-CCGCTAGCATTACCCTCC-3′.

## Results

### Expression of Zebrafish Fibrinogen

**Figure 1:**
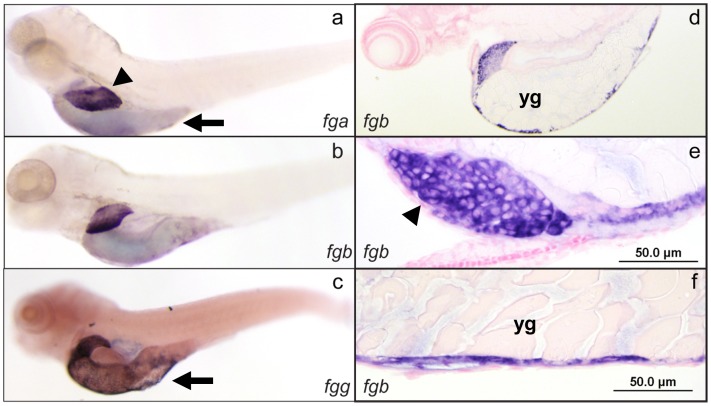
*In situ* hybridization of zebrafish fibrinogen genes demonstrates liver and yolk sac specific expression. Whole mount *in situ* hybridization was performed using antisense mRNA probes complementary to the *fga*, *fgb*, and *fgg* mRNAs (a, b, and c respectively) at 5 dpf. The expression patterns were identical, with high levels in the liver and yolk sac. Individual experiments showed variability in yolk sac expression for all 3 genes, e.g. lower (a, b) and higher (c) levels of expression. Sections through *fgb* probed larvae confirmed that liver expression was in hepatocytes, while the yolk sac staining was in the outer cellular layer (d–f). Arrowheads indicate the liver, while arrows point to the yolk sac; yg, yolk granules. Sections of *fga* and *fgg* probed larvae demonstrated the same pattern, while *in situ* hybridization with probes generated from the sense strand did not show any signal (not shown).

The zebrafish liver bud is first distinguishable approximately 48 hours post fertilization (hpf) to the left of midline, overlying the yolk sac [36]. From 60–72 hpf (2.5–3 dpf) the liver grows until it has reached its final dimensions relative to the larva. Outgrowth completes by 5 dpf, leaving a bilobed organ with a left lobe that is larger than the right [36]. Expression patterns of the three fibrinogen chain mRNAs, (*fga*, *fgb*, and *fgg*) were examined in the developing zebrafish at 3 and 5 dpf. All three are expressed throughout the liver at days 3 and 5 (5 dpf shown in Fig. 1). Thin sections demonstrated that expression is localized to hepatocytes (Fig. 1d, e). Additionally, expression is seen in the yolk sac on day 5 (Fig. 1a–f). Sections through the yolk sac revealed that expression is localized to the outer syncytial layer (Fig. 1f).

### Fibrinogen is Incorporated into Induced Zebrafish Thrombi

**Figure 2:**
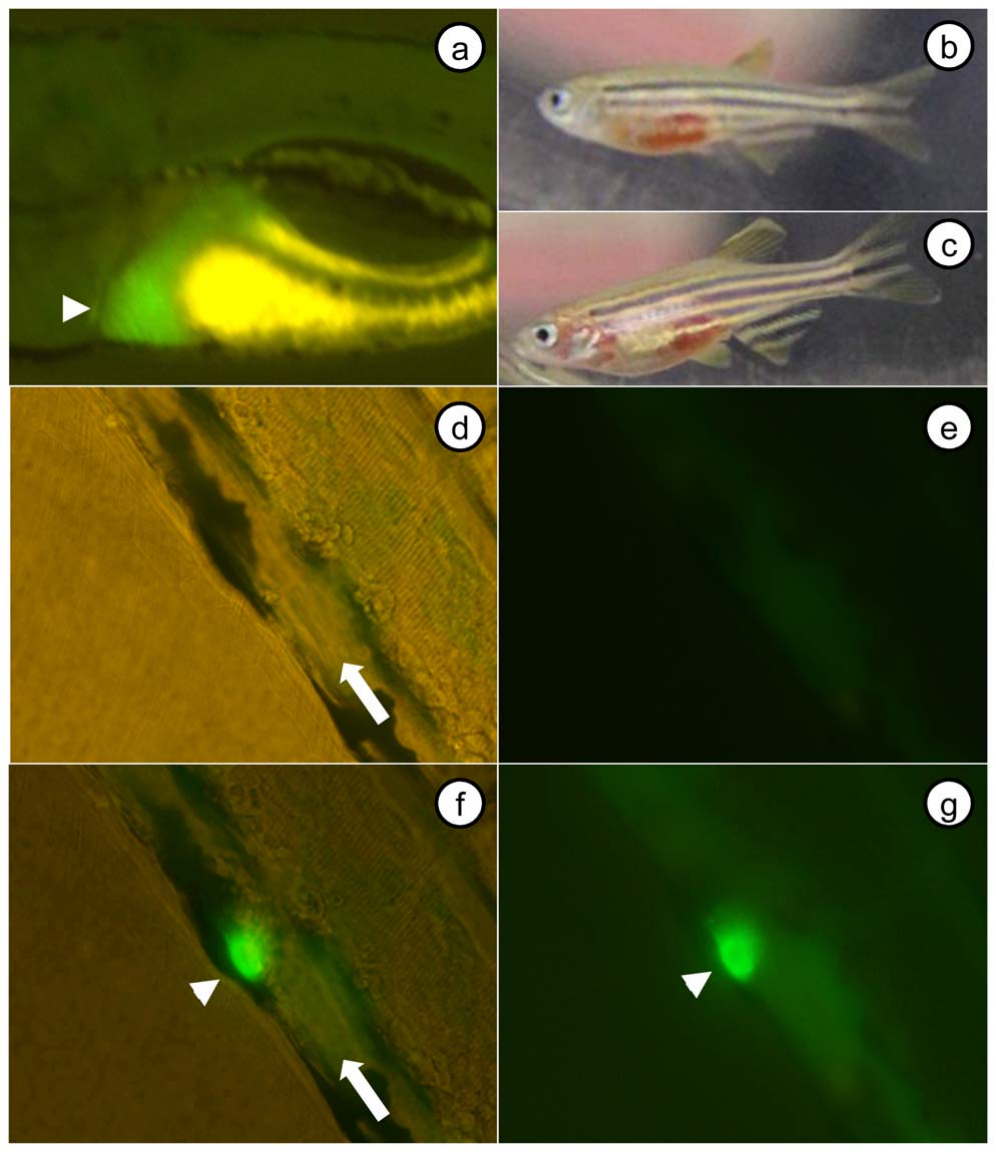
Liver specific expression of *fgb-egfp* results in incorporation of fusion protein into an induced thrombus. A *fabp10-fgb-egfp* transgenic line of zebrafish was produced, and F1 offspring analyzed. (a) Expression was liver specific, as demonstrated by the region of green fluorescence in a 5 dpf larva (yellow signal is yolk sac autofluorescence). (b, c) F1 adult fish displayed overt hemorrhage, suggestive of potential interference with coagulation secondary to dysfibrinogenemia. Pictures of the PCV prior to (d, brightfield+fluorescence; e, fluorescence only), and after (f, brightfield+fluorescence; g, fluorescence only) laser injury (f, g were prior to complete occlusion). The arrowhead in a indicates liver, arrows in d, f indicate PCV and direction of flow, and arrowheads in f, g point to the induced thrombus.

It has been demonstrated previously that induced injury by laser ablation of endothelial cells initiates thrombus formation in zebrafish larvae [21]. In order to evaluate whether fibrinogen is a constituent of this thrombus formation *in vivo*, we constructed an *fgb-egfp* fusion gene containing full length *fgb* cDNA and placed it under control of the liver specific *fabp10* promoter [31]. As previously shown [31], this promoter has the ability to drive specific expression of the *fgb-egfp* transgene in the liver (Fig. 2a). Fluorescence is not visible in the intravascular space in unperturbed embryos or larvae (Fig. 2d, e). However, endothelial injury of the posterior cardinal vein (PCV) at 5 dpf resulted in the formation of fluorescent intravascular thrombi (Fig. 2f, g, Movie S1). Among adult F1 transgenic offspring, 17 of 39 displayed visible hemorrhage (Fig. 2b, c), suggesting a potential dysfibrinogenemic effect of the fusion protein.

### Fibrinogen Knockdown by Morpholinos Results in Widespread Hemorrhage

**Figure 3:**
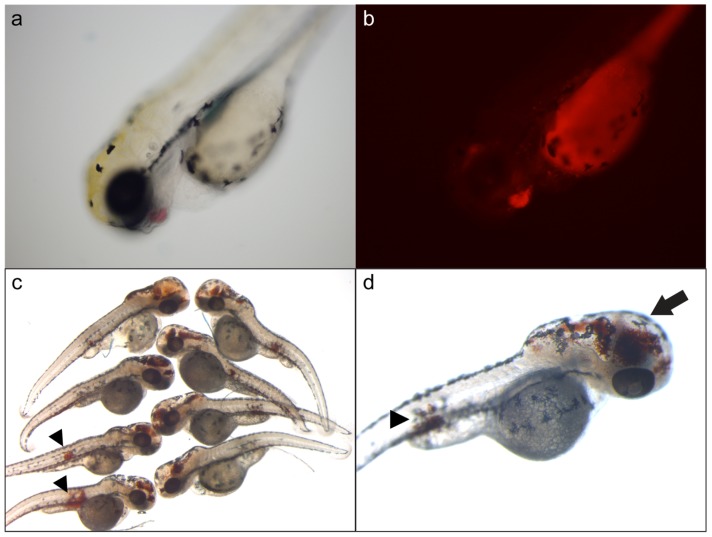
Morpholino knockdown of *fga*, *fgb*, and *fgg* results in intracranial and intramuscular hemorrhage. Single cell embryos were injected with 1*fga*, *fgb*, and *fgg* splice blocking MOs, and analyzed at 3 dpf. Larvae displayed what appeared to be hemorrhage, primarily intracranial, which was confirmed by two methods. (a) Example of an injected *gata1-dsred* transgenic larva with apparent hemorrhage in the facial region, confirmed under fluorescence (b). Note that the yolk sac displays autofluorescence. o-dianisidine staining detects hemoglobin, which identified sites of hemorrhage (c, d). These sites included intracranial hemorrhage with secondary hydrocephalus (arrow in d), as well as apparent intramuscular hemorrhage (arrowheads in c, d).

**Figure 4:**
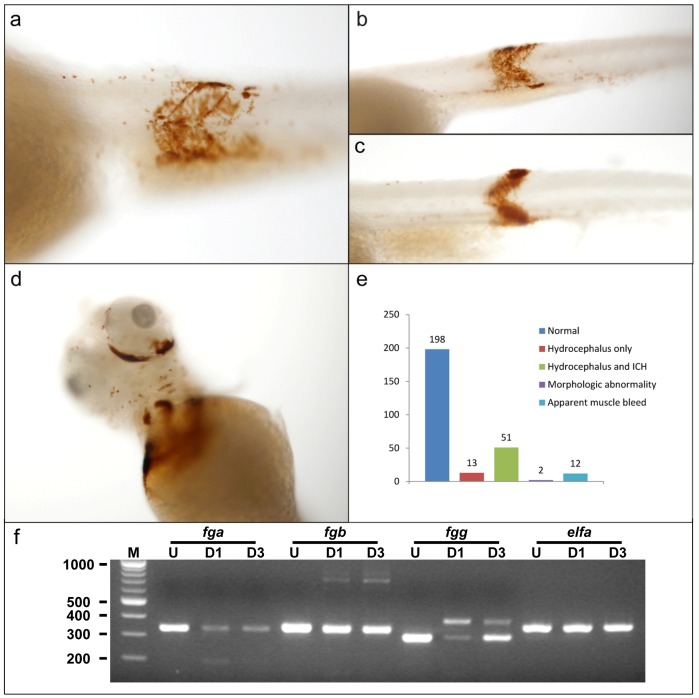
Additional sites of hemorrhage in fibrinogen morphants. Other sites of hemorrhage were visible in morpholino-injected larvae. Single cell embryos were injected with 1 nl of a mixture containing a final concentration of 0.5 mM of each of *fga*, *fgb*, and *fgg* splice blocking MOs, and then analyzed at 3 dpf by o-dianisidine staining. (a–c) Examples of morphants with intramuscular hemorrhage. Arrow shaped muscle segments are clearly highlighted by o-dianisidine staining, indicating hemorrhage. (d) Example of orbital hemorrhage. (e) Larvae were evaluated under a low power dissecting microscope and scored in the categories described. The 12 larvae with apparent muscle bleeds (as shown in a–c) also had hydrocephalus with or without ICH (intracranial hemorrhage). Control larvae displayed no abnormalities. (f) RT-PCR analysis of larvae after MO injection. Total mRNA was prepared from pools of 10 embryos or larvae that were uninjected (U), 1 day post injection (D1), or 3 days post injection (D3). RT-PCR was performed using primers in exons flanking the target splice donor sites. The quantity of parental cDNA product was diminished after injection, and altered splicing lead to additional products that were reduced (*fga*) or increased (*fgb* and *fgg*) in size, consistent with MO knockdown. *elfa* (elongation factor I alpha) was included as control for mRNA integrity. M, molecular weight marker with sizes in base pairs.

In order to confirm that clotting in zebrafish is dependent on fibrin formation, we designed splice-blocking morpholino oligonucleotides (MOs) to all three zebrafish fibrinogen chains. These were injected into zebrafish embryos followed by analysis at 3 dpf using fluorescence in *gata1-dsred* transgenic fish [27] (Fig. 3a, b) or by o-dianisidine staining for hemoglobin (Fig. 3c, d, 4a–d). The fractions of larvae with hemorrhage were 5%, 2%, and 1% for *fga*, *fgb*, and *fgg* injected singly, respectively (data not shown). However, when all three MOs were co-injected, there was a synergistic increase in the frequency of hemorrhage to nearly 20% (Fig. 4e). We observed various hemorrhagic phenotypes, which were primarily intracranial (Fig. 3c, d). Many larvae developed what appeared to be secondary hydrocephalus with or without intracranial hemorrhage (Fig. 3c, d). Additionally, a significant proportion displayed intramuscular hemorrhage (Fig. 3c, d arrowheads, and Fig. 4a–c). Figures 4a–c demonstrate muscle segment hematomas in several larvae, and other observed sites of hemorrhage included the peri-orbital space (Fig. 4d).

We confirmed that the MOs specifically targeted the fibrinogen mRNAs by performing RT-PCR on injected embryos and larvae at 1 and 3 dpf, respectively. Knockdown was partial for all 3 targets with evidence of alternative splicing as well as quantitative reduction of the parental cDNA products (Fig. 4f). In the cases of *fga* and *fgg* the effects were more prominent on day 1, and faded by day 3. There was lesser reduction of the *fgb* cDNA product, but the effect was maintained through 3 dpf. *elfa* (elongation factor I alpha) was included as control for integrity of the mRNA [37] and showed consistent levels across all time points.

## Discussion

Conversion of fibrinogen to fibrin is a required step for the eventual formation of a stable clot. Patients with acquired and congenital deficiencies of fibrinogen are at risk for various forms of hemorrhage [3], [4]. In patients with congenital deficiencies of fibrinogen, bleeding is often mucocutaneous, while intracranial hemorrhage is the most common cause of lethality, and musculoskeletal bleeding is less frequent than hemophilia [3], [4]. In this study we have phenocopied human hypofibrinogenemia by knockdown of zebrafish fibrinogen. This was confirmed both by o-dianisidine staining as well as the use of a fluorescent transgene that labels erythrocytes, both leveraging the transparent nature of zebrafish larvae. While reduction of a single fibrinogen chain resulted in a low frequency of hemorrhagic events, knockdown of all three demonstrated a synergistic effect on the incidence of observed phenotypes. These data are somewhat in contrast to afibrinogenemic mice and humans, in which loss of a single chain completely abolishes the production of circulating fibrinogen [3], [38], [39]. However, the MOs failed to effect complete loss of target mRNA, the strongest reduction was at 1 dpf, and levels began to normalize by 3 dpf, features which are known disadvantages of MO technology [40]. Together these points likely explain why the percentage of larvae displaying hemorrhage was low, as well as the synergy of combined knockdown. As in human patients, the most frequent major phenotype of affected larvae was intracranial hemorrhage, with a lesser number displaying the equivalent of intramuscular hematomas.

As a vertebrate model, the zebrafish has many advantageous features that distinguish it from mammalian organisms. Embryonic and larval development are external, greatly simplifying phenotypic screening. A number of forward genetic screens with chemical mutagenesis have produced significant insight into cardiogenesis, vasculogenesis and angiogenesis [20], [41]–[43]. We used *in situ* hybridization to show that all three fibrinogen chains are expressed at 3 and 5 dpf in the developing yolk sac and liver. Sections at 5 dpf demonstrated hepatocyte specific and outer yolk sac layer expression. This complements previous analysis demonstrating expression up to 48 hpf, as well as at 4 and 6 dpf [25]. These results are concordant with sites of fibrinogen expression in mammalian models, including liver (rat and mouse, [44], [45]) and yolk sac (rat, [45]).

In this study, we demonstrated that we could achieve tissue specific expression of an Fgb-eGFP fusion protein with the hepatocyte specific *fabp10* promoter. While expression was not strong enough to be visible in the intravascular space, induced endothelial injury of the PCV resulted in the development of green fluorescent thrombi. This demonstrates that the tagged zebrafish fibrinogen beta chain is able to incorporate into a developing clot, and this can be directly visualized with simple microscopy. In the future, this process could be visualized using confocal microscopy to perform similar intravital studies previously accomplished in the mouse [46]. Interestingly, despite a reasonable frequency of transient transgenesis in F0 embryos, the rate of germline transmission was surprisingly low despite use of the Tol2 system [30], and adults displayed obvious hemorrhage. The structure and function of fibrinogen has been well described [47], [48], and this phenotype suggests interference from the eGFP tag. The C-terminus of human FGB is a component of the D region, and charge dependent interactions with the E region of adjacent fibrinogen monomers are required for linear polymerization. Destabilization of this interaction could interfere with polymerization and subsequent factor XIII crosslinking, causing the formation of an unstable fibrin clot, or producing fibrin with increased sensitivity to fibrinolysis. We hypothesize that the C-terminal eGFP, which is 258 amino acids in length, might interfere with these processes through a variety of mechanisms, including steric hindrance, alteration of charge, or improper folding. Indeed there are a significant number of pathological mutations at the 3′ end of human *FGB* [49], and thus the incorporation of fibrinogen containing Fgb-eGFP into the crosslinked fibrin mesh may be simulating dys- or afibrinogenemia. One example of a pathologic C-terminal mutation is the common FGB substitution R448L, which alters D region conformation thus affecting fibrin structure, clot rigidity and fibrinolysis [50]. This work complements previous studies demonstrating conservation of zebrafish and human fibrinogen. Regulatory elements in the human fibrinogen gene cluster have been characterized based on their ability to drive hepatic expression in zebrafish larvae [25], [51], [52]. Cationic dendrimers known to induce coagulopathy *in vivo* were shown to accelerate clot formation *in vitro* by actions directly on fibrinogen in the absence of thrombin [53]. Injection of dendrimers into zebrafish larvae resulted in complete intravascular occlusion, presumably through similar mechanisms. We have now shown that zebrafish fibrinogen is incorporated into an induced thrombus and may simulate dysfibrinogenemia, while knockdown results in hemorrhage characteristic of human patients with hypo- and afibrinogenemia. Taken together, these data are strong evidence for a high degree of functional conservation between humans and zebrafish, demonstrating the utility of this vertebrate model.

## Supporting Information

Movie S1 **Real-time** ***in vivo*** **imaging of the incorporation of Fgb-eGFP into an induced venous thrombus.** Laser mediated endothelial injury of the posterior cardinal vein (PCV) in *fabp10-fgb-egfp* transgenic larvae at 5 dpf demonstrated formation of fluorescent intravascular thrombi. The specimen was visualized solely under fluorescence until 00∶20, and then visible light was added.
(MP4)
